# Contagious Venereal Disease Amongst Horses in Kent County, Canada

**Published:** 1890-03

**Authors:** Peter H. Bryce


### THE JOURNAL
OF
### COMPARATIVE MEDICINE AND VETERINARY ARCHIVES.

VOL. XI. MARCH 1890. NO. 3.

### CONTAGIOUS VENEREAL DISEASE AMONGST HORSES IN KENT COUNTY, ONTARIO.

BY PETER H. BRYCE, M.A., M.D.

A. The biological and pathological characters of the disease.

B. The mode of propagation, extent and severity of the disease.

C. Recommendations deemed necessary for dealing with the disease.

*A. The biological and pathological characters of the disease.*

There is, perhaps, no single class of disease amongst horses, of which the exact relationships and differentiations between the different varieties of phenomena have been more under discussion or more debated than that of venereal diseases; and after a careful perusal of the veterinary literature of the subject I have arrived at the conclusion that at present there is no published view of which I am aware which is at all adequate to describe the etiological (causational) features of the disease which has appeared in Kent. This being the case, I have endeavored by a reference to the extended studies which have been made of venereal diseases in man, and by an investigation more especially of the biological facts relating to these diseases, to set forth in some systematic manner the principal biological and pathological phenomena of venereal diseases in horses so far as they have appeared in this county.

This has seemed to me all the more necessary inasmuch as those veterinarians who have had personally to deal with this outbreak have very divergent views both as to the genesis, clinical affinities and serious characters of the disease; while further, it is proper that any who may dissent from any recommendations made in this report should be in a position to become fully acquainted with the views held by me regarding the nature of the disease, as supplying the arguments upon which subsequent recommendations are based.

Amongst men venereal disease has existed from the earliest time of which medical science has any record; but so late as the beginning of this century no differentiation of its varieties had been satisfactorily made. During the past forty years a differentiation likely to hold has been made, by which syphilis is definitely separated from gonorrhœa, while certain ulcerative phenomena which may become superadded to both have been relegated more or less satisfactorily to their own place, as adjuncts to or causative of the inflammatory processes which may be present.

Veterinary literature presents the same evident tendency to differentiations; but owing, fortunately, to the differences in circumstances by which, as will be seen in the sworn evidence of our most prominent veterinarians, very few opportunities have occurred in English-speaking countries, and notably in Canada, for studying equine venereal disease, no recent work of an advanced biological character seems to have been done in this field, and hence we are impelled to make a comparative study of these diseases in man and horses, with the hope of adding to the more exact knowledge of the subject.

The first phenomena in any venereal disease which are likely to be observed, either by a surgeon or the owner of an animal, are those due to *inflammation* and *septic fever.* If we gain a clear idea of this, which is in no sense different from any other inflammation or phlegmon, except in the matter of the region infected, the subsequent phenomena will seem capable of explanation in their proper order. Such an inflammation is due to the inoculation, at some stage in the progress of the disease, into the tissues, in this case the vaginal mucous membrane, or the soft external parts, of the bacterium of suppuration now known as the *staphylococcus pyogenes aureus.* Hundreds of experiments have shown this to be true, while thousands of serious surgical operations are now yearly performed with an almost perfect certainty that if *asepticism* can be preserved, that is, the non-introduction of the *pus-making* micro-organism, no inflammatory action will follow.

When inoculation does take place, either accidentally, as when a wound is made in the integument or the mucous membrane, or by experimental inoculation, what happens is that the micrococci rapidly multiply, utilizing blood clot or blood plasms for their nourishment.

"This fermentative decomposition produces from its very beginning certain alkaloids or chemical, extremely poisonous substances, the *ptomaines* that are very diffusible. By dint of this diffusibility, the adjacent vasomotor nerves at once come under their toxic influence, as the result of which their strong dilatation ensues, which becomes manifest in the shape of an *active hyperæmia*, '*rubor*.'

"The blood passing through the adjacent arterioles and capillaries seems also to become altered; the red blood-corpuscles become packed and finally stagnate in the capillaries and smaller arteries. The walls of these vessels, including the veins, lose their impermeability, and a number of white and often red blood-corpuscles emigrate into the surrounding tissues, densely infiltrating their interstices, thus producing the characteristic swelling, '*turgor*.'

"As a consequence of the increased blood supply, possibly also of the active chemical process, a marked increase of the local temperature is observed, '*calor*.' And, if we add that pain. '*dolor*,' of the parts thus affected is never absent, we have completed the classical cycle of the four cardinal symptoms of inflammation—'*rubor*, *calor*, *turgor*, *dolor*.' "—Gerster, pp. 176–9, "Aseptic and Antiseptic Surgery.

The progress of the inflammation to the stage of suppuration, or the formation of pus and abscesses, is so well set forth in the following translation of a critical review by E. Metchnikoff, in *Annales de l' Institut Pasteur*, p. 133, Mars, 1889: "Sur le Role et le Sort du Staphylococcus Aureus Dans le Peau,"—(On the Role and the Fate of the *Staphylococcus Aureus* in the Skin)—that I reproduce it in full as presenting in a condensed form the most advanced views on the subject based upon direct experiment.

According to the researches of M. Hohnfeldt, made in the laboratory and under the direction of M. Baumgarten, the staphylococcus, immediately after its introduction in the subcutaneous tissue of rabbits, sets up marked lesions, which become manifest four hours after the experiment has been made, under the form of an agglomeration of leucocytes, some of which hold within themselves staphylococci. Parallel with the multiplication of these latter, the number of leucocytes considerably increases, and there is produced a leucocytary infiltration which subsequently becomes transformed into a true cutaneous abscess.

The cells which constitute this abscess are for the greater part multinuclear leucocytes, with the nucleolus strongly tinged with aniline colors, and are undoubtedly derived from the leucocytes of the blood, immigrated to the place of invasion. Many of these cells contain considerable quantities of staphylococci, which become colored similarly with those outside the leucocytes. M. Hohnfeldt does not doubt that the microbes mentioned have been *englobed*, or rather have entered spontaneously into the interior of leucocytes, of which there soon remains only a species of granular residuum.

In following the development of the abscess, we can prove that it is filled by great quantities of free staphylococci, isolated or united in groups, and by migratory cells, more or less degenerated, and reduced to a granular condition.

In its peripheral part, the abscess is surrounded with a limiting capsule, which, in short, forms quite slowly (toward the tenth day after infection). One then perceives a karyokynetic division in the nuclei of the cells of the conjunctival tissue and of its newly-formed epithelioidal cells, which indicates the purely reparative character of this phenomenon.

As the abscess, arriving at the periphery of the skin, opens generally towards the exterior, the organism frees itself at the same time from the pathogenic staphylococci, as also of the dead and more or less decomposed cells. M. Hohnfeldt does not attribute in the destruction of the staphylococci any role to the motile cells, and thinks, on the contrary, that these phagocytes present a very favorable medium for the life and multiplication of pyogenic microbes.

*En resume*, M. Hohnfeldt confirms the opinion of his teacher, M. Baumgarten, that the theory of phagocytes is found to be in disaccord with the pathological phenomena of the formation of abscesses. Such is not the opinion of M. Ribbert, who for his part has undertaken elaborate researches on the same subject.

He has followed from the outset the march of infection, in introducing small quantities of staphylococci under the skin of rabbits. While M. Hohnfeldt injected half a cubic centimetre in a thick emulsion, M. Ribbert limited himself to introducing into a slit in the skin several millimetres long a scalpel dipped in an emulsion of staphylococci. Following this lesion, small abscesses sometimes formed, but in most cases the affection did not go beyond a slight hyperæmia and a tumefaction around the wound. The inoculation thus produced with minute quantities of staphylococci was always followed by a healing, complete at the end of some days, and accomplished notably by the aid of phagocytes. These latter, under the form of leucocytes, with a multiple nucleus and with fixed cells of conjunctival tissue, agglomerate in the place of invasion directly from the first day, and englobe all the staphylococci, which soon show evident signs of degeneration; they take color in an incomplete manner, and show unequal dimensions. Around the inflamed part, and later in the same part, one observes the karyokynetic reparative phenomena, in a great number of nuclei of tissue cells, both conjunctival and epithelial.

The introduction of large quantities of staphylococci is followed, according to M. Ribbert, by phenomena of another kind. The inflammatory action of leucocytes, as also the production of abscesses, becomes much more considerable, and a great number of phagocytes perish under the influence of microbes. These latter, collected in groups more or less large, are found for the most part outside of the small multinuclear leucocytes, and show evident signs of death, and become the prey of large macrophagic cells (springing from the fixed cells of the conjunctival tissue).

"In these cases M. Ribbert attributes a restraining role to isolated phagocytes, but admits that the totality of immigrant leucocytes forms around the masses of staphylococci an impregnable barrier. The agglomerated microbes then perish, not under the direct influence of the phagocytes, but rather because of indirect action, notably following the want of oxygen and nourishment, as well as from the presence in considerable quantities of toxic products of the same bacteria.

"M. Ribbert expresses himself of the opinion that between phagocytosis properly called and the enveiopment of microbic masses by a leucocytary mantle there occur all the intermediary passages in such a way that the two phenomena cannot be rigorously separated. On associating myself with this view I seize the occasion to recall to our readers who would interest themselves in the subject of phagocytes that in my introductory article (published in the review of M. Clauss, in 1883, *vide* p. 156) I have made known the two modifications of the processes indicated above. 'The role of amœboid mes dermic cells,' said I, 'consists in devouring the parts of the organism, and become useless, as well as the foreign bodies, or rather in the cases where this solution is not possible at least serve to envelop them and to retain them in place.'

"At the end of his memoir, M. Ribbert opens the question of the influence of liquid parts of the organism on staphylococci. After the introduction of these microbes into the parts of the skin inflamed by the action of iodine, the staphylococci multiply in the œdematous liquid of the tissues with such a freedom that neither the isolated phagocytes, nor the leucocytes united in a mass, can form a serious obstacle to their invasion. The staphylococci multiply in a surprising manner, and, not finding sufficient resistance, provoke the necrosis of the parts invaded; but they are not, however, in a state to break through the barrier formed at the limit of the sound tissue by the leucocytes arrived at this place. One well sees that in these cases the liquids accumulated by the inflammation present conditions very favorable to the increase of the invasive microbe, and that healing is only possible with the aid of the formation of a thick layer of leucocytes and by the desquamation of the necrosed part.

"In accord with M. Hohnfeldt on the source of the cells of pus and on the role especially reparative of the fixed cells of the conjunctival tissue, M. Ribbert differs totally, as we have seen, from the pupil of Baumgarten on the subject of the fate of the staphylococci introduced into the skin of rabbits."

From inoculation through the various stages, from hyperæmia to the formation of pus, the progress of this inflammation has been traced ; and we have now to examine more in detail the inflammation set up in this venereal disease. The most recent writers on the subject seem to be still greatly divided in their opinions as to whether in any urethritis or vaginitis any other agency than that of staphylococci is necessary for the production of the phe nomena even in a gonorrhœa.

J. W. White, M.D., Professor of Genito-urinary Diseases, in the University of Pennsylvania, is positive in his view that gonorrhœa differs in no essential from an ordinary urethritis, while on the other hand Gerster, following Bumm, assumes it to be proved that gonorrhœa is due to a specific microbe termed the gonococcus, but adds the following important statement in a note (p. 301) :

"The presence of various micro-organisms, aside from the gonococcus, in recent and chronic urethral discharges seems to point to the fact that *most cases of urethritis represent a mixed form of bacterial infection.* There is no doubt that the inoculation of pyogenic microbes into a gonorrhœally affected mucous membrane forms an important element in determining the intensity and perniciousness of some very bad cases. This assumption is also made in accordance with the theory of the development of metastases, notably of gonorrhœal rheumatism. Bumm is very reserved in regard to the acceptance of Kammerer's investigations, who found gonococci in recent effusions produced during an attack of gonorrhœal rheumatism. On the other hand, we know that rheumatic attacks are occasionally provoked by an instrumental examination of a patient afflicted with 'simple' or 'catarrhal' or 'traumatic' urethritis in which the absence of gonococci is indisputable. Finally, the frequent presence of simple pyogenic organisms in rheumatic effusions is generally accepted. It seems then that pus-generating organisms play an important part in cases of gonorrhœic and non-gonorrhœic urethritis, and that the metastatic processes complicating urethral inflammations are mostly chargeable *to them and not to the presence of gonococci.*"

Again, in the very recent monograph on "Blenorrhœa of the Sexual Organs," by Dr. Ernest Finger, University of Vienna, the whole subject of gonorrhœa is fully treated. A summation is therein given of the conclusions based upon experiment of the most prominent bacteriologists who, since Neisser's reported discovery, in 1879, of the gonococcus, have investigated the subject. The evidence might be said to be almost evenly balanced *pro* and *con ;* but the author, after several pages of references to different experimenters, says :—"Finally, Bumm's treatise adduced abundant material and incontrovertible proof of the virulent character of the micro-organisms," and thereafter proceeds to describe the gonococcus.

Now it is generally conceded that the clinical features of gonorrhœa are in no single point distinctive from those of urethritis due to (*a*) leucorrhœa, (*b*) pus from an abscess, (*c*) endometritis, (*d*) acid vaginal discharges, (*e*) powerful injections, (*f*) gravel, (*g*) catheterism. May it therefore not be assumed that in every instance of urethritis we may explain the inoculation of the virus which sets up the inflammation to be due to the staphylococcus pyogenes, as we know it must be in all cases other than those where the assumed gonococcus may have been the microbe inoculated?

There are, further, other facts bearing upon the problem which must be mentioned in this connection. In a by no means infrequent number of cases of gonorrhœa, where a low vitality of tissue is present owing to excesses, etc., and where uncleanly habits exist, there is not only a free formation of pus in the secretions of the mucous tract, but the tissues are also further broken down by abscesses, and ragged-edged ulcers appear, with the same appearances as are seen in what are known as chancroids, at one time supposed to be indicative of syphilitic lesions. In Prof. White's opinion any differences of appearance in gonorrhœal ulcers are not differences in kind but in degree. They are discrete, confluent or rodent, according as a less or more irritating or corrosive pus is present. In the latter instance the intense inflammation may so extend as to cause a lymphangitis, a bubo, cowperitis, prostatitis or cystitis, and in females vulvo-vaginal abscess, due to its spread to the Bartholini glands. Chronic effects, seen in mucous patches common in the chancroid inoculation associated with syphilis, may further be present, due to the oozing puriform secretions from the ulcerations. What has been stated as belonging to the ulcerations which from time to time appear in gonorrhœas may be repeated as being the characteristics peculiar to the chancroid or so-called simple venereal ulcer in contradistinction to the true chancre. Now it is interesting to note that at least as early as 1867 the character of the chancroid, as due to pus inoculation, was being established. Pick, of Vienna, Kraus and Reder made inoculations from the pustules of scabies, pemphigus and acne; while Wigglesmith, an American student in Zeissl's, Vienna, clinic carried to several generations auto-inoculations from acne pustules. Similarly in Paris, successful inoculations from the ecthyma pustules on typhoid patients were made by MM. Leclerc and Vigla.

Summing up, Dr. Sturgis, of New York, concludes, first, that auto-inoculation is not peculiar to simple venereal ulcers; second, that simple pus, under certain circumstances, is capable of inoculation, and third, that this inoculation is due partially to irritative inflammation, affecting the tissues, and partially to the character of the tissues themselves. Thus, if a non-venereal pustule is irritated and excited to action, its pus he believes will be inoculable. Indeed it was found in the experiments of Kraus and Reder that the inoculations in order to be successful had to be of recently formed pus—*i. e.*, from new abscesses.

Manifestly, therefore, whether we are to assume the inflammatory irritation of the tissues to be due to an invasive microbe, called the gonococcus, to a laceration of tissue set up by a too violent coitus, to instruments, or to the erosive or caustic action of drugs, whereby an hyperæmia of the tissues is produced, it becomes apparent that the destructive agent by which the inflammation or phlegmon is continued is in all cases due to the staphylococcus pyogenes aureus.

In view of the investigations which have been made by Neisser and others since his time, with the view of proving a distinct and specific micrococcus as the cause of gonorrhœa, we may advert briefly to their bearing upon the points which we have been considering. According to Bumm the micrococcus of gonorrhœa has a peculiar invasive quality, penetrating rapidly into the submucous tissues, there setting up an inflammation which gives to the staphylococcus an easy access to the tissues where its peculiar effects are developed in the formation of pus. Apparently, however, the only distinctive character, according to the above writer, apart from a supposed peculiarity of form, due to fission, in the multiplication of the microbe is the fact *that its presence within the pus corpuscle is more or less constant.* "The *favorite location* of the gonococci found in the urethral secretion is within the pus corpuscles. This peculiarity belongs exclusively to the coccus of gonorrhœa, detected by Neisser in 1879, and represents its most important characteristic."

Now we have seen in the experiments of Hohnfeldt how the very creation of pus corpuscles is in a large measure due to the presence within the leucocytes of the blood of the destructive staphylococci of pus. To use the exact words of Metchnikoff: "*Beaucoup de ces cellules contiennent des quantités considerables de staphylocoques, qui se colorent aussi bien que ceux qui se trouvent hors des leucocytes.*" Ribbert and Metchnikoff refer to the same fact in speaking of the destructive action of the leucocytes (phagocytes) on these pathogenic microbes.

Speaking for ourselves after a review of the facts, biological and clinical, bearing on this most important matter, we have to confess our belief that no single fact has yet been produced which is sufficient to demonstrate the existence of a specific gonococcus apart from the staphylococcus pyogenes; while on the other hand innumerable clinical facts and such biological ones as we have already referred to seem to stamp the latter as the essential cause of gonorrhœa. In support of this conclusion we have:

(a) The clinical evidence of the various exciting causes of urethritis already referred to; (b) the localized hyperæmia of the erotic state, called in animals *œstrum* or rut. This hyperæmia as the condition of inoculation is on all hands recognized. A congested pharynx conditions the inoculation of the diphtheritic microbe, a part, *e. g.*, the bursa of the knee, constantly irritated by pressure, or a breast hyperæmic with the milk flow after parturition conditions the formation of abscess, while the extensive area of the vaginal mucous membrane, hyperæmic under the condition stated above, all equally attest this truth; (c) laceration of parts, whether extensive or slight, as must frequently occur during coitus in mares, whereby with the attendant hyperæmia, inoculation of the staphylococcus from pus, whether from an unclean stallion or from pus corpuscles already present in the leucorrhœa of a mare, must almost inevitably take place; (d) hyperæmia, set up by septicæmic, or putrefactive microbes as in the puerperal state, making inoculation with the staphylococcus readily possible.[1]

[1] The following brief abstract from a report of the Société Centrâle de Médecine Vétérinaire, Paris, is of interest:

Séance du 10 Octobre, 1889; President, M. Chauveau, in chair.

1st. Report on equine venereal diseases by M. Laquerièrre. This work of M. Blaise, military veterinarian, confirms what has already been established by observers:—

1° The exanthem of coition is nothing else than equine variola, the horse-pox, situate on the genitals.

2° *La dourine* (equine syphilis) of the horse is a disease analogous to syphilis in man and not identical; exactly in the same way as the rot in sheep is analogous to small-pox in man but is not identical with it.

M. Fournier has found the most complete analogy from the point in view of symptoms between certain wounds in *dourine* and the parchment chancre, or by the mucous patches, in the following cases: *La dourine* is never completely cured; it seems contagious only during the time in which the wounds of the genitals are observed.

A difficulty may here be raised against this view from the fact that in old mares with chronic leucorrhœal discharges and in stallions or men with a gleety discharge no auto-infection in many instances takes place. It must be remembered, however, that in all these instances there has been or is a more or less chronic congestion of the mucous membrane in which interstitial infiltration has taken place, causing a hyperplasia or excessive formation of connective tissue with a low condition of vitality of the mucous membrane and excessive glandular secretion and shedding of mucous cells. But further than this, Zeller has very recently discovered that in a chronic catarrh the cylindrical epithelium of the uterus becomes transformed into stratified pavement epithelium to the extent that the most superficial layer may become cornified. The mucous membrane takes on an epidermic aspect. Mr. Schuchardt has shown that the same thing occurs in chronic ozœna in the Schneiderian membrane.—*Journal des Sciences Scientifiques, May, 1889.*

Such congestions create, with their lessened arterial blood supply and acrid secretions, a condition itself antagonistic to the free multiplication of microbes, as has been shown in the case of inoculations with pus from chronic abscesses ; but which, nevertheless, continue a state of affairs, only requiring some such exciting circumstances as the excitement of œstrum in mares or of repeated coition in stallions, or excesses in men to light up anew an inflammation, owing to the free multiplication of staphylococci. An analogous condition exists in scrofulous children or others with enlarged tonsils and chronic pharyngitis, whereby they become subject to repeated attacks of ulcerative tonsillitis or diphtheria.

Summing up, then, all the facts, we are forced to the conclusion that the inflammatory condition with its concomitant phenomena, which has distinguished the outbreak of veneral disease in the horses of Kent County, is one dependent primarily upon a localized hyperæmia due to various causes, but mostly due to the erotic state ; that while it may be possible that a specific microbe—a gonococcus—may have been the immediate cause of the inflammation, yet the staphylococcus pyogenes aureus has caused, as in every other case of phlegmon or suppuration, the clinical phenomena characteristic of the disease ; and further, that the history of inoculation with the pyogenic microbe *per se* supplies all the data necessary for an explanation of the causation of the outbreak ; and that the fact of the disease assuming an epidemic form this year is as explainable on the supposition that some one or more initial cases of active phlegmon existed, during which fresh virulent pus was developed—providing its inherent power to inoculate be accepted as proven from the many facts given—as upon the theory that another microbe called the gonococcus was present in some one or more initial cases. The contagiousness of the disease appears to me to be equally explainable on either hypothesis.

B. *The clinical features, mode of propagation, extent and severity of the disease.*

In the preceding section I have, in discussing the relationships of venereal diseases in the matter of their causation, incidentally made reference to the clinical phenomena of the disease under review. It will be proper, however, to here briefly set them forth in some regular order as they have come either under the observation of those veterinarians, whose evidence is given in the appendix, or of myself.

*Symptoms in the mare.*—After a mare has been bred to a horse in a condition to communicate the disease, there appears commonly within a week localized irritability of the genital tract with increased frequency of micturition, apparenlty accompanied by pain and straining as evidences of local discomfort. Soon a vaginal discharge of a leucorrhœal character appears, in which pus corpuscles soon appear collected in flocculi by means of the albuminoid secretion. In less acute cases this discharge may be the first sign which has attracted the owner to the fact of there being anything wrong. Examinaticn by a veterinarian *per speculum* or by separating the lips of the vulva shows a swollen and intensely hyperæmic mucous membrane, varying in intensity in different parts and in different cases. Especially is there congestion and swelling in the region of the clitoris, which often is the seat of ulcerations. These beginning as blebs soon appear at different parts of the vagina and rapidly break down, appearing as ragged ulcers loaded with pus cells. These ulcers may remain single, or several enlarging become concrete. The inflammatory action may extend to the uterus, which is especially true in those instances where mares are bred, as is frequently the case, within eight days or a fortnight after foaling. The discharges bathing external parts irritate the vulva especially, but where profuse they affect also the epidermis on the underside of the root of the tail and the folds of the soft skin, down even as far as the mammæ. Denudation of pigment is usually the first result of this irritation, and this is followed at many points by inoculation with the virus, presumably the streptococcus pyogenes, and ulcers form at first singly, but in many instances rapidly extending, form large ulcers which in the folds of soft skin may extend for several inches. This was notably the case in the Dauphin mare, which was seen by myself, as also in a Wallaceburg mare reported at a quite recent date. The mare of William White, also seen by myself, showed several large ragged ulcers externally. All these external parts, notably the vulva, become swollen, at times very greatly. The swelling in one case seen by myself and referred to in the evidence of W. B. Rowe, V.S., Ridgetown, was very extensive on the right vulva, and became intensely painful, causing the mare great distress, and only relieved by a free incision, which gave escape to the pus of a large abscess, presumably due to extension of the inoculation along the duct of a vulvo-vaginal gland. The meatus of the urethra becomes involved in the inflammation and a urethritis with all the distressing symptoms of straining and frequent micturition is present. How extensive this local inflammation and ulceration may become is illustrated in the case of the Sieveright mare, where, whatever laceration might have been present as the result of parturition, the phlegmon became so intense after being bred to "Rock and Rye" that straining created rupture of the recto-vaginal tissues, making the destruction of the mare necessary.

With such extended local infection and inflammation it is natural to suppose that general symptoms, such as a frequent pulse and elevated temperature, should supervene. The evidence of J. Steen, V.S., especially, proves that such was the case. In some instances several degrees above the normal were attained. Some of the cases seemed to present other evidences of constitutional disturbances, such as discharges from both nose and eyes, and here and there enlargement of the cervical lymphatics.

Some degree of spinal tenderness was noticed in several cases, and in the fatal case which came under Mr. Steen's observation, being that of Wm. Clark, of Fletcher, this symptom became very marked. The degree of this was slight, however, in most cases that progressed favorably. In such cases as the latter it was probably the result of reflex irritation rather than systemic poisoning. When absorption of pus has set up what is comparable to gonorrhœal rheumatism, the spinal tenderness may fairly be considered as of pyæmic origin, and, we must suppose, due to the ptomaine poisons produced either in the mucous membrane or in the lymphatics crowded with streptococci.

In the fatal and other severe cases, as in the history of the Dauphin mare, the Wallaceburg mare, and the McKinlay mare, pyæmic infection became general and abscesses developed in glands or along the course of the lymphatic vessels, to in some instances a notable extent. Swelling of the limbs and other evidences of lymphatic engorgement occurred, as might have been expected.

*Symptoms in the stallion.*—What has been said with regard to the local appearances of the disease in the mare may, as far as local conformation of organs makes comparison possible, be said of the disease in the horse. The part primarily exposed is the external portion of the penis and that of the sheath in contact with it. The denudation of pigment here becomes noticeable, and the first minute, round denuded areas are succeeded by others, running along for several weeks. In addition to this sign of the disease we are likely to have more positive evidences of infection in a urethritis appearing within three or four to eight or ten days after exposure. The external appearances of this may be comparatively slight, nothing more appearing than a congested appearance with a slight muco-purulent discharge. The inflammation may, however, extend upwards to the membranous portion of the urethra, as also along the seminal ducts and to cowper's glands. The swollen, congested condition of this mucous tract produces heated, painful and frequent micturition, which same condition induces at once a local excitement and venereal desire, with a difficulty in completing the act of coition through the pain produced. The period of scalding and difficult micturition having passed either in the progress of the disease or owing to treatment, it leaves the mucous membrane in a venously congested state and with excessive secretion. As stated by Mr. Steen, external examination, especially after micturition, may show no secretion of a purulent character in such cases, but if catheterization is practised a muco-purulent discharge is likely to follow the withdrawal of the catheter, it being produced and retained especially in the membranous portion of the urethra and the ducts of the various glands. These may be said to be the signs common to an ordinary case in the stallion. Few cases, however, came under observation in which, owing to the more or less continued use of the stallion, the condition did not become subacute and the conditions common to gleet continue. Swelling and sensitiveness of the urethra partially disappeared and nothing more than an undue moisture of the meatus was present to observation, but the capacity for communicating the disease to mares covered by these stallions remained. That it would be to a mare covered in the morning that the danger of inoculation was greatest is evident for obvious reasons.

Remarks on the denudation of pigment on the prepuce and upper parts of penis, as also the inner side of sheath, have been made. There is no doubt but that such parts, especially in horses not carefully cleansed by washing after a service may, while showing no serious swelling, become a source of continued supply of the virus, which by simple contact becomes applied to the mucous membrane of a mare, causing an inoculation, if in a mare after foaling, with swollen, congested or lacerated parts, or in a young mare through lacerations owing to the violence of the coitus. The facts stated by Mr. Steen in relation to Mr. W. White's stallion and mare especially illustrate this point. In this case the stallion was the carrier of the disease at a period when no symptoms had appeared in him—in fact two days after serving a diseased mare, supposed to have been *cured*.

In some cases, however, as will be noticed by the evidence, the external parts become more seriously involved, the denuded areas in some instances breaking down into small ulcers, notably on the preputial ring, at times coalescing. I assume the stallion at St. Clair, Mich., stated by Mr. McGregor, of Courtright, to have been so badly affected with rodent ulcers, to have been an extreme example of such a case. The extent of swelling of the sheath or the prepuce will be in proportion as it becomes involved in this inflammatory and ulcerative process, due to the direct inoculation or extension of the swelling to it from other affected parts.

The testicles, as would be expected, hang down abnormally owing to relaxation of the cremaster muscles from loss of tone in nerves supplying the part, although occasionally reflex irritation may cause them to unduly contract and the testicles will then be drawn up close to the ring. Atrophy, when the inflammatory action had extended to the testicle, as in subacute cases, would be expected, and an interesting fact may here be noted that the horse "Henry Abraham," which, as stated by Mr. Bogart, V.S., died from rupture in the beginning of the season of 1889, had been the cause of an outbreak of the disease late in the Summer of 1888.

The general symptoms in the stallion are of a somewhat negative character. The animal seems usually more or less sluggish, the eye dull, the appetite variable, and while excessive excitement is likely to be present during the stage of active hyperæmia, yet when this has passed off the general *malaise* shows itself in a varying indifference to the presence of mares and a failure to accomplish coition. Spinal tenderness, as might be expected, occasionally may be found in this as in other states of constitutional disturbance. The secretions from the eyes and nose may be increased in amount and in viscidity. Should absorption from extended ulcerations take place, the same lymphatic swellings and systemic consequences may follow as did follow in several mares. That such states are serious in that they predispose to other diseases may be seen from the fact that the horse "Young Conqueror" is said to have died from a pleurisy during the time when affected by the disease, but subsequent to injury by a stake. Whether or not the stallion "Rock and Rye" had any dregs of the old disease remaining may, from his history be doubted, but it is interesting to note that he is dead—I understand from inflammation of the bowels.

With the constitutional effects produced by inoculation with the virus of the disease, as increased temperature, disturbed nutrition, local inflammation with excessive secretion and formation of pus, emaciation will take place in the degree that the systemic effects are severe and continued. With the diffuse lymphangitis which, as has been observed, appears in many intances, defective elimination of effete matters follows, and should decisive measures both hygienic and curative not be adopted, I do not see how it is possible to expect any result other than that the pus poisoning should become a permanent one, and the fatal results, which all observers of outbreaks of *maladie de coit* in horses have recorded, be the logical sequence of just such a set of clinical phenomena as have been recorded. In the several fatal cases of the disease observed by veterinarians during this outbreak of the past Summer, the later symptoms in one, viz., the Clark mare, affected especially the nervous system. Congestion and extreme sensibility of the spinal cord existed, ending in tottering gait, inability to stand, owing to posterior paralysis, the animal being relieved from suffering by being killed. Other cases as the Dauphin mare, the Sieveright mare, the Wallaceburg mare, and the McKinlay mare, showed more prominently the lymphatic phenomena peculiar to pyæmia. The localized swellings, abscesses, emaciation, staring coat, all were present, having in all but one case the subacute or chronic, and too frequently fatal, effects of pyæmia.

Such, then, are the clinical facts belonging to the outbreak in Kent and surrounding districts. In view of the diverse views which have here and there been expressed with regard to its clinical affinities to venereal disease as it has appeared elsewhere in this country, or has been described in the literature of outbreaks which have occurred elsewhere, we propose to briefly compare it with outbreaks of venereal disease in other countries, as set forth in the literature of the subject.

[TO BE CONTINUED.]

